# Supplementary material for: Targeting MK2-Driven Stromal Senescence Sensitizes Breast Cancer Bone Metastasis to Immunotherapy
Source: bioRxiv. 2026 Oct 28:2026.10.26.761990. Preprint. [Version 1] doi: 10.64898/2026.10.26.761990 (PMC13733941; doi:10.64898/2026.10.26.761990)
Supplement: Supplementary file 1 [file NIHPP2026.10.26.761990V1-supplement-1.pdf]

bioRxiv preprint doi: https://doi.org/10.64898/2026.10.26.761990; this version posted October 28, 2026. The copyright holder for this preprint (which was not certified by peer review) is the author/funder, who has granted bioRxiv a license to display the preprint in perpetuity. It is made available under a CC-BY 4.0 International license.

REAGENT	SOURCE	IDENTIFIER	DILUTION/DOSAGE
**Neutralization/Blocking Antibodies**			
*InVivo*MAb anti-mouse CD4 (Clone: GK1.5)	BioXcell	Cat# BE0003-1	500µg/mouse (first dose); 250ug/mouse (following doses every 4 days)
*InVivo*MAb anti-mouse CD8a (Clone: 2.43)	BioXcell	Cat# BE0061	500µg/mouse (first dose); 250ug/mouse (following doses every 4 days)
*InVivo*MAb anti-mouse CSF1 (Clone: 5A1)	BioXcell	Cat# BE0061	1mg/mouse (first dose); 500ug/mouse (following doses every 4 days)
*InVivo*MAb anti-mouse CTL4 (Clone: UC10-4F10-11)	BioXcell	Cat# BE0032	200µg every 3 days
*InVivo*MAb anti-mouse PD-1 (Clone: RMP1-14)	BioXcell	Cat# BE0146	200µg every 3 days
**Agonist Antibodies**			
*InVivo*MAb anti-mouse OX40 (Clone: OX-86)	BioXcell	Cat# BE0031	200ug/mouse (every 4 days - 3 doses total)
**Other Reagents**			
Clodronate Liposomes	Liposoma	Cat# C-010	200ul/mouse (every 4 days)
Fixable Viability Dye eFluor-780	Invitrogen	Cat# 65-0865-14	1:1000
Dnase I Recombinant	Sigma	Cat# 4536282001	2U/mL
Collagenase from Clostridium histolyticm	Sigma	Cat# C0130-5G	2mg/mL
AEC (3-amino-9-ethylcarbazole) chromogenic substrate	ABCAM	Cat# ab64252	-
Hematoxylin	DAKO	Cat# S3301	-
**Flow Cytometry Antibodies**			
FITC Anti-mouse CD4 (Clone: RM4.4)	Thermo Scientific	Cat# 11-0043-82	1:200
BUV395 Anti-mouse CD8a (Clone: 53-6.7)	BD Biosciences	Cat# 563786	1:200
BV510 Anti-mouse CD8a (Clone: 53-6.7)	Thermo Scientific	Cat# MA5-29682	1:200
Pacific Blue Anti-mouse CD45 (Clone: 30-F11)	Biolegend	Cat#103126	1:400
BV785 Anti-mouse CD45 (Clone: 30-F11)	Biolegend	Cat# 103149	1:400
APC Anti-mouse CD3e (Clone: 145-2C11)	Thermo Scientific	Cat# 17-0031-63	1:200
BV711 Anti-mouse CD3e	BD Biosciences	Cat# 563123	1:200

bioRxiv preprint doi: https://doi.org/10.64898/2026.10.26.761990; this version posted October 28, 2026. The copyright holder for this preprint (which was not certified by peer review) is the author/funder, who has granted bioRxiv a license to display the preprint in perpetuity. It is made available under a CC-BY 4.0 International license.

(Clone: 145-2C11)			
BV605 Anti-mouse CD62L (Clone: MEL-14)	Biolegend	Cat# 104438	1:50
eFluor450 Anti-mouse CD44 (Clone: IM7)	Thermo Scientific	Cat# 48-0441-82	1:400
APC Anti-mouse CD19 (Clone: eBio1D3)	Thermo Scientific	Cat# 17-0193-82	1:200
Alexa Fluor 700 Anti-mouse CD11b (Clone: M1/70)	Thermo Scientific	Cat# 56-0112-82	1:400
APC-eFluor780 Anti-mouse CD11c (Clone: N418)	Thermo Scientific	Cat# 47-0114-82	1:50
eFluor450 Anti-mouse MHCII I-A/I-E (Clone: M5/114.15.2)	Thermo Scientific	Cat# 48-5321-80	1:400
FITC Anti-mouse Ly-6G (Clone: 1A8)	Biolegend	Cat# 127605	1:400
PerCP-Cy5.5 Anti-mouse Ly-6C (Clone: HK1.4)	Thermo Scientific	Cat# 45-5932-82	1:400
PE-Cy5 Anti-mouse F4/80 (Clone: BM8)	Thermo Scientific	Cat# 15-4801-82	1:400
PE Anti-mouse PD-1 (Clone: J43)	Thermo Scientific	Cat# 12-9985-82	1:100
**IHC Antibodies**			
Mouse CDKN2A	Abcam	Cat# ab241543	1:10 *discontinued cell supernatant version
Human CDKN2A	Abcam	Cat# ab108349	1:400
Mouse aSMA	Cell Signaling Technology	Cat# 19245	1:00
Human/mouse aSMA	Abcam	Cat# ab5694	1:200
Mouse PDGFRa	Cell Signaling Technology	Cat# 3174S	1:500
Mouse pan-Cytokeratin	Novus Biologicals	Cat# NBP2-29429	1:1000
Human pan-Cytokeratin	Novus Biologicals	Cat# NBP2-29429	1:1000
Mouse CD68	Cell Signaling Technology	Cat# 97778T	1:1000
Mouse/human ARG1 (Clone: D4E3M)	Cell Signaling Technology	Cat# 93668	1:100
Mouse CD4 (Clone: 4SM95)	Thermo Scientific	Cat# 13-9766-82	1:100
Mouse CD4 (Clone: EPR19514)	Abcam	Cat# ab183685	1:200
Mouse CD8a (Clone: 4SM16)	Thermo Scientific	Cat# 14-0195-82	1:200
Mouse MHCII I-A/I-E (Clone: M5/114.15.2)	Thermo Scientific	Cat# 48-5321-82	1:500

bioRxiv preprint doi: https://doi.org/10.64898/2026.10.26.761990; this version posted October 28, 2026. The copyright holder for this preprint (which was not certified by peer review) is the author/funder, who has granted bioRxiv a license to display the preprint in perpetuity. It is made available under a CC-BY 4.0 International license.

Mouse/Human pHsp27	Cell Signaling Technology	Cat# 9709	1:100 (AEC mIHC); 1:200 (DAB)
Mouse pMK2	Cell Signaling Technology	Cat# 3007	1:400
Rabbit anti-rat IgG	Vector	Cat# AI-4001-.5	1:500
Goat anti-rabbit IgG Fab fragment	Jackson ImmunoResearch	Cat# 111-007-003	1:25
**IF antibodies**			
Mouse CDKN2A	Invitrogen	Cat# PA1-46220	1:100
Goat anti-rabbit IgG Alexa Fluor 594	Thermo Scientifc	Cat# A-11037	1:500
**Western Blot Antibodies**			
Mouse/human MK2	Cell Signaling Technology	Cat#	1:1000
Mouse/human pMK2	Cell Signaling Technology	Cat# 9218S	1:1000
Mouse GAPDH (Clone: GAPDH-71.1)	Sigma Aldrich	Cat# G8795	1:2000

Table 1. Table SI. List of reagents used in this work. The list includes neutralizing and blocking antibodies, flow cytometry antibodies, western blot, immunohistochemical and immunofluorescence antibodies, and other reagents with their respective source, identifier and dilution or dose employed.
