# Supplementary material for: Targeting MK2-Driven Stromal Senescence Sensitizes Breast Cancer Bone Metastasis to Immunotherapy
Source: bioRxiv. 2026 Oct 28:2026.10.26.761990. Preprint. [Version 1] doi: 10.64898/2026.10.26.761990 (PMC13733941; doi:10.64898/2026.10.26.761990)
Supplement: Supplementary file 4 [file media-3.pdf]

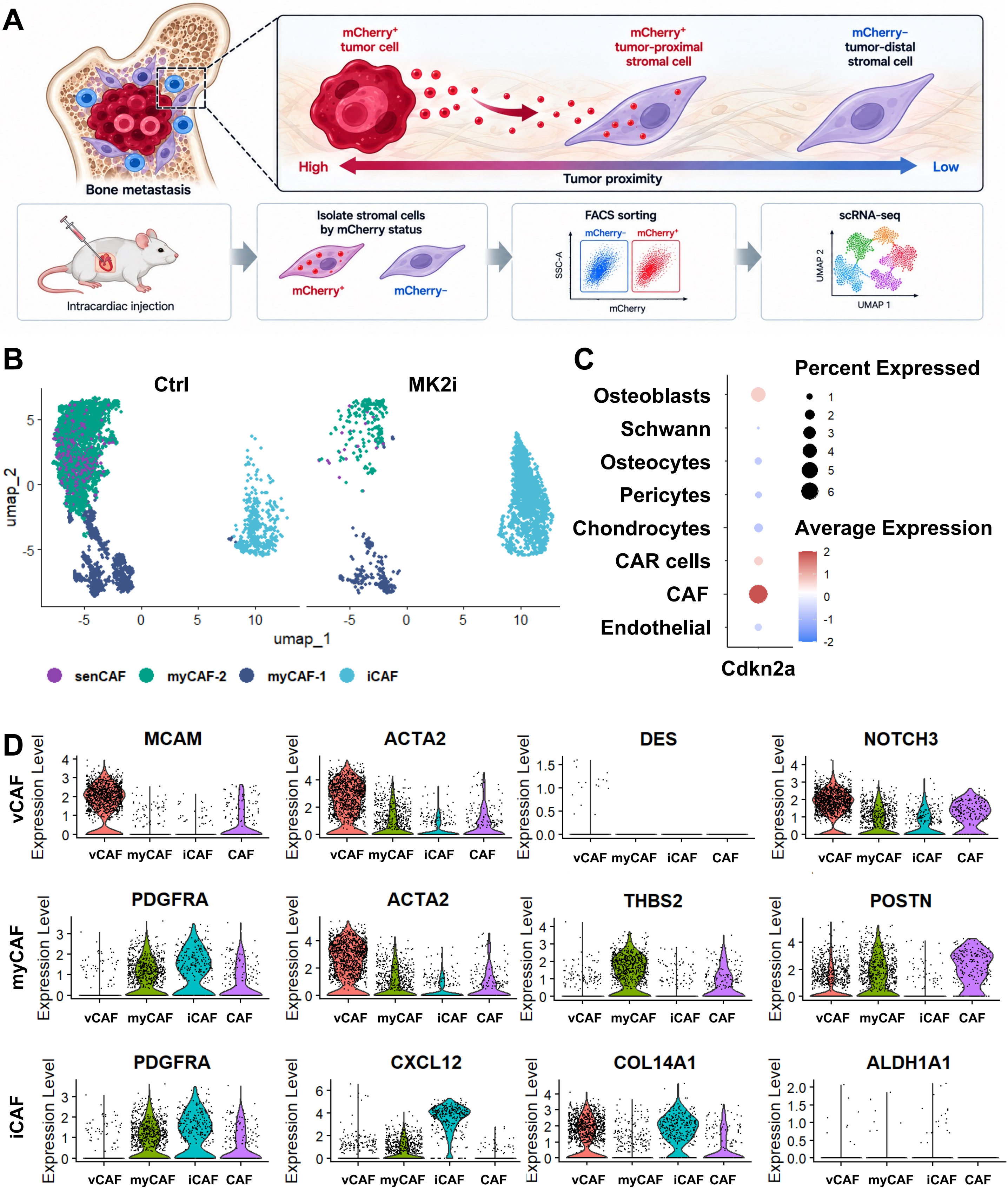

F. S3, Ramalho-Oliveira *et al.*

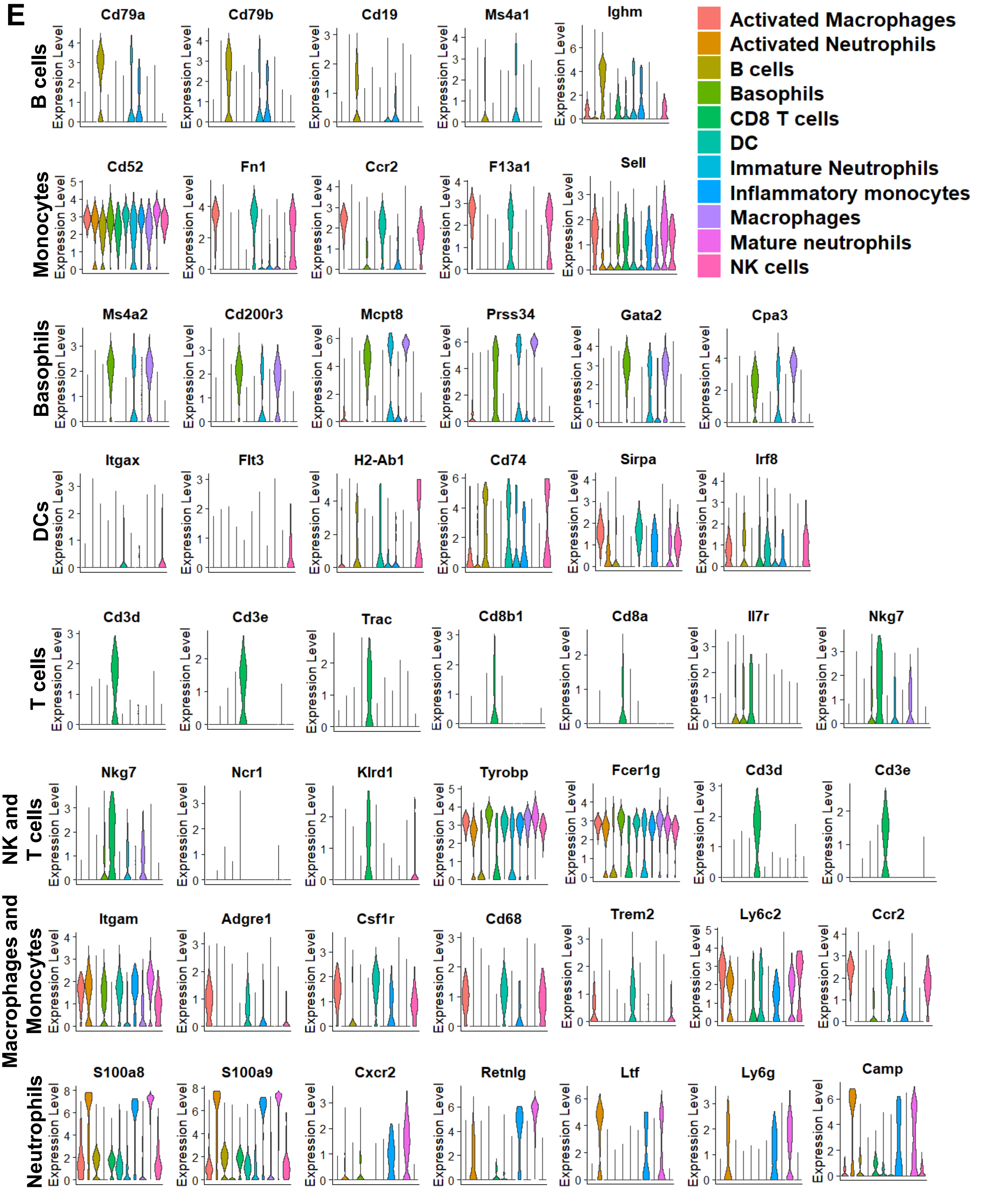

F. S3E, Ramalho-Oliviera *et al.*

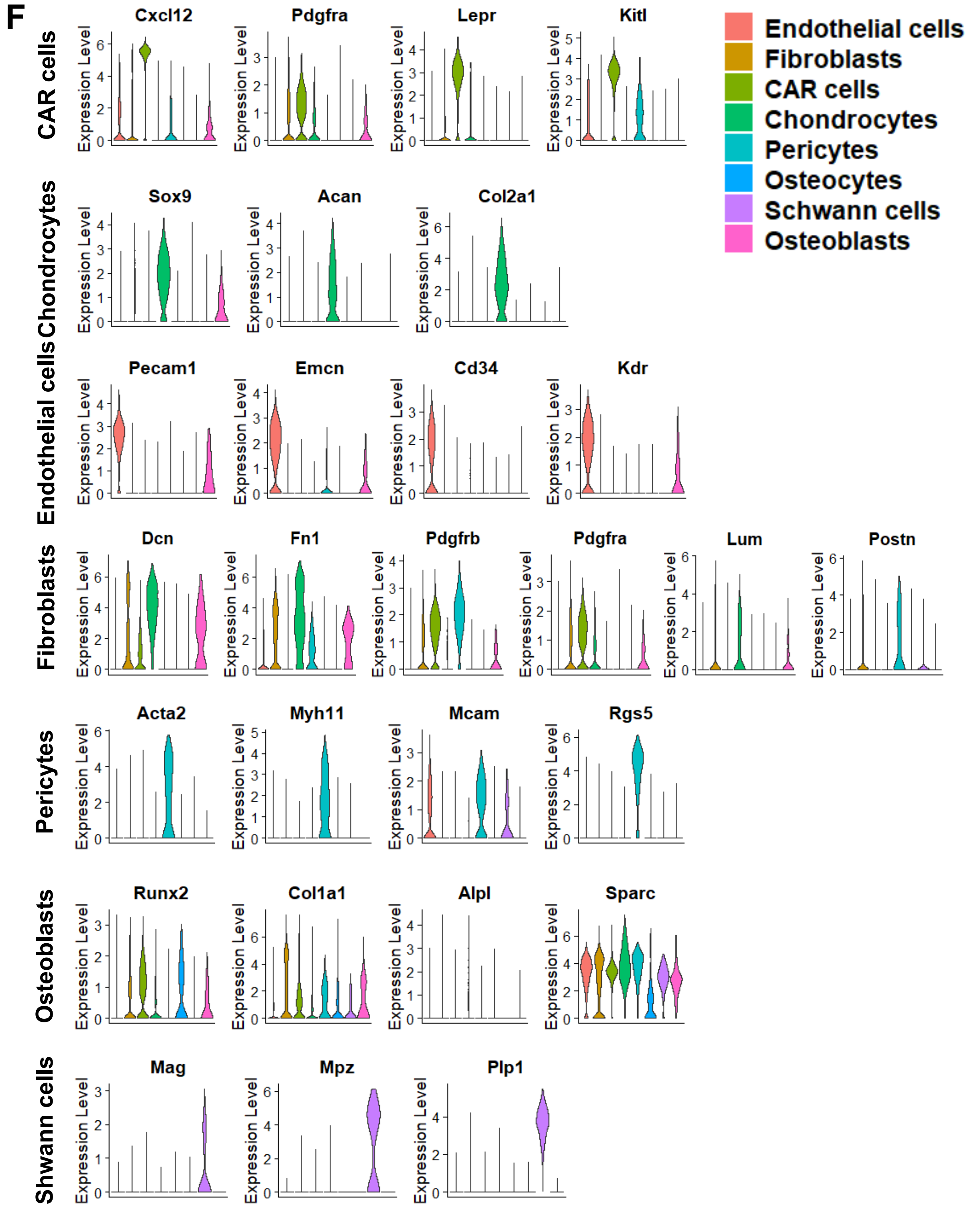

F. S3F, Ramalho-Oliviera *et al.*
